# Current real-life survival after segmentectomy for lung cancer

**DOI:** 10.3389/fsurg.2026.1923394

**Published:** 2026-08-26

**Authors:** Xavier Cansouline, Abdoul-Khadry Gassama, Abdelhakim Elmraki, Béatrice Lipan, Damien Sizaret, Delphine Carmier, Antoine Legras

**Affiliations:** 1 Thoracic Surgery Department, Tours University Hospital, Tours, France; 2 N2C UMR 1069, University of Tours, INSERM, Tours, France; 3 Department of Pathology, Tours University Hospital, Tours, France; 4 Department of Pneumology, Tours University Hospital, Tours, France

**Keywords:** lung cancer, oncologic resection, postoperative recurrence, postoperative survival, segmentectomy, sublobar resection

## Abstract

**Introduction:**

Segmentectomy for non-small cell lung cancer (NSCLC) is increasingly performed for small-sized lesions. However, prognostic factors associated with poor outcomes after this lung-sparing oncologic surgery remain uncertain.

**Methods:**

We conducted a single-center retrospective study including 100 consecutive patients who underwent single or multiple segmentectomies for NSCLC in real-world practice.

**Results:**

In univariate analyses, compromise segmentectomy (HR 3.52; *p* = 0.005), complex segmentectomy (HR 2.56; *p* = 0.020), SUVmax ≥ 6 (HR 2.86; *p* = 0.017), and thoracotomy (HR 2.62; *p* = 0.020) were associated with worse overall survival. In multivariable analysis, compromise segmentectomy remained associated with worse overall survival (HR 3.47; *p* = 0.026). Regarding recurrence, thoracotomy was associated with poorer disease-free survival (HR 2.77; *p* = 0.040).

**Conclusion:**

In this real-world cohort, compromise segmentectomy identified a high-risk subgroup with impaired overall survival, probably reflecting baseline frailty and comorbidity rather than a clearly demonstrated increase in recurrence. These findings support careful preoperative selection and counseling of patients considered for lung-sparing resection outside standard indications.

## 1 Introduction

In localized-stage non-small cell lung cancer (NSCLC), the standard treatment is curative-intent surgery consisting of an anatomical resection of the pulmonary parenchyma with thorough lymph node dissection. Historically, tumor resection was performed as lobectomy; however, technical advances and accumulating scientific evidence have allowed thoracic surgeons to limit the extent of resection to one or more segmentectomies in selected patients. This increasingly widespread practice has recently been incorporated into international guidelines (1). These recommendations apply to two main categories: (1) peripheral solid or part-solid nodules smaller than 2 cm with cN0 status on PET/CT imaging; and (2) pure ground-glass nodules measuring up to 3 cm.

Considering these changes in clinical practice, it is necessary to clarify risk factors for recurrence and mortality following sublobar oncologic surgery in real-world practice, particularly among patients undergoing compromise segmentectomy outside standard indications.

## 2 Materials and methods

### 2.1 Study design and population

We conducted a single-center retrospective study at the CHRU of Tours (France) including all consecutive patients who underwent single or multiple segmentectomies for NSCLC between January 1, 2019, and December 31, 2022. Clinical records were prospectively included in the French Epithor database, a government-recognized clinical database financially supported by the French National Cancer Institute and the French Society of Thoracic and Cardiovascular Surgery for data-quality monitoring. Patient consent was obtained for entry into the database, and patients were informed that these anonymized data could be used for research purposes. The French National Commission for Data Protection approved the use of this database (CNIL No. 809833). This study was conducted according to the principles of the Declaration of Helsinki. Patients with incomplete R1 or R2 resection were excluded. Patient informed consent for this retrospective analysis was waived because of the retrospective and anonymized nature of the study.

All included patients underwent 18F-FDG PET-CT and thin-slice chest CT to determine cTNM stage, lesion location, and consolidation-to-tumor ratio (C/T ratio, defined as the ratio of the solid component of the lesion to its maximum diameter) (2). Intentional segmentectomy was defined as a segmentectomy performed according to standard eligibility criteria: (1) a peripheral solid or part-solid lesion smaller than 2 cm with cN0 status on PET/CT imaging; or (2) a pure ground-glass lesion measuring up to 3 cm. Compromise segmentectomy was defined as segmentectomy performed outside these standard criteria in patients considered unsuitable for lobectomy, including patients with lesions larger than 2 cm, bifocal tumors, impaired FEV1 and/or DLCO and/or VO2max, significant comorbidities, or lesions spanning more than one lobe. Patients with neuroendocrine tumors of the carcinoid type were excluded.

### 2.2 Surgical procedure

Segmentectomies were performed via thoracotomy, video-assisted thoracoscopic surgery (VATS), or video-assisted mini-thoracotomy. Video-assisted mini-thoracotomy was defined as an incision of approximately 7 cm without rib spreading, using both direct vision and video-assisted thoracoscopic guidance. For survival analyses, VATS and video-assisted mini-thoracotomy were pooled as video-assisted approaches, whereas open thoracotomy was analyzed separately. Fissures and intersegmental planes were divided using staplers after verification of resection margins. Systematic lymph node dissection, either radical or lobe-specific, was performed in all cases. When intraoperative lymph node involvement was suspected, frozen-section analysis was requested, and lobectomy was performed if N+ disease was confirmed.

A simple segmentectomy was defined as involving a single intersegmental plane, such as apicodorsal (S1-S2), right anterior (S3R), lingular (S4-S5), culmen (S1-S2-S3L), superior segment (S6), or basal pyramid (S7-S8-S9-S10) segmentectomy. A complex segmentectomy was defined as involving two or more intersegmental planes, such as segmentectomies within the basal pyramid, apical (S1), dorsal (S2), or left anterior (S3L) segments (3).

### 2.3 Postoperative follow-up

All patients in this series were seen in the thoracic surgery outpatient clinic one month after hospital discharge. They subsequently underwent CT-based follow-up and clinical evaluation by their pulmonologist every six months for two years, then annually for five years. Patient outcomes were assessed in 2025 either through medical correspondence or direct telephone interviews with patients. Recurrence was classified into three categories: local recurrence at the bronchial stump or within the operated lobe; regional recurrence in another ipsilateral lobe, hilar or mediastinal lymph node recurrence, or recurrence within the ipsilateral pleural cavity; and distant recurrence, including contralateral recurrence or recurrence outside the thorax.

### 2.4 Statistical analysis

The primary endpoint was disease-free survival (DFS), with overall survival (OS) as the secondary endpoint. Analyses were performed using R software and initially consisted of univariate Cox regression analyses of clinical and intraoperative variables. Additional analyses were conducted according to resection margin status (R0 vs. Run), segmentectomy complexity (simple vs. complex), C/T ratio (cutoff 0.5) (4), segmentectomy intent (intentional vs. compromise), and SUVmax (cutoff 6) (5). Uncertain resection (Run) was defined according to the IASLC classification as insufficient lymph node dissection, positivity of the highest mediastinal lymph node, carcinoma *in situ* at the bronchial margin, or positive pleural cytology (6). Variables found to be statistically significant in univariate analyses were subsequently included in multivariable Cox regression models. Kaplan–Meier survival curves were generated for surgical approach, segmentectomy intent, resection complexity, and SUVmax. Given the limited number of events, multivariable results were interpreted cautiously and considered exploratory.

## 3 Results

We included 100 patients whose clinical characteristics are reported in Table 1. Most patients had adenocarcinoma (84%) and pathological stage 0-I disease (78%). Surgery was mainly performed by video-assisted mini-thoracotomy (70%). In the survival analyses, 79 patients were classified in the video-assisted approach group and 21 in the thoracotomy group. Compromise segmentectomy represented 19% of cases, and R0 resection was obtained in 60% of patients. The mean follow-up duration was 43.7 months. Seventeen patients experienced recurrence, and 22 patients died, among whom 8 deaths were cancer-related. Among the recurrences, there were 6 local recurrences, 3 locoregional recurrences, 5 metastatic recurrences, and 3 new primary cancers. The mean time to recurrence was 20 months, whereas the mean time to death was 27.2 months.

**Table 1:** Clinical characteristics of the cohort.

Variable	Value, *n* (%) or median (IQR)
Clinical characteristics	
Male/female sex	60/40
Age, years, median (IQR)	70 (61–74)
BMI, median (IQR)	26.62 (23.2–29.1)
WHO performance status 0–1/2–3	96/4 (96%/4%)
Respiratory function	
Dyspnea 0–1/2–4	98/2 (98%/2%)
Smoker	75 (75%)
Pack-years, median (IQR)	37 (20–50)
Preoperative FEV1, % predicted, median (IQR)	83 (71–100)
Predicted postoperative FEV1, % predicted, median (IQR)	73 (64–85)
Preoperative FEV1/FVC ratio, median (IQR)	74 (60–80)
Preoperative DLCO, % predicted, median (IQR)	70 (58–88)
SUVmax, median (IQR)	3.2 (2.1–5.8)
C/T ratio, median (IQR)	0.85 (0.5–1)
cTNM	
T1	84 (84%)
T2	5 (5%)
T3-T4	11 (11%)
N0/N1–2	95/5 (95%/5%)
Surgical procedure	
Video-assisted mini-thoracotomy	70 (70%)
VATS	9 (9%)
Thoracotomy	21 (21%)
Compromise segmentectomy	19 (19%)
Operative time, minutes, median (IQR)	184 (150–204)
Hospital length of stay, days, median (IQR)	3 (2–6)
Maximum Numeric Rating Scale for pain, median (IQR)	5 (3–6)
Histology	
Adenocarcinoma	84 (84%)
Squamous cell carcinoma	16 (16%)
pTNM	
T1	77 (77%)
T2	9 (9%)
T3-T4	14 (14%)
N0/N1–2	90/10 (90%/10%)
R0/Run	60/40 (60%/40%)
Stage 0-I	78 (78%)
Stage II	12 (12%)
Stage III	10 (10%)
Recurrence	17 (17%)
Death	22 (22%)

IQR, interquartile range; BMI, body mass index; WHO, World Health Organization; FEV1, forced expiratory volume in one second; DLCO, diffusing capacity for carbon monoxide; SUVmax, maximum standardized uptake value; C/T ratio, consolidation-to-tumor ratio; VATS, video-assisted thoracoscopic surgery; Run: uncertain resection.

The results of overall survival and disease-free survival in univariate Cox regression analysis are presented in Table 2.

**Table 2:** Univariate results for disease-free survival and overall survival.

Characteristic	DFS HR	DFS 95% CI	DFS *p*-value	OS HR	OS 95% CI	OS *p*-value
Sex (male)	3.28	[0.94–11.46]	0.060	4.75	[1.40–16.08]	0.012
Age	1.01	[0.95–1.08]	0.770	1.05	[0.99–1.11]	0.089
BMI	1.03	[0.93–1.15]	0.540	0.91	[0.83–1.01]	0.068
WHO performance status	1.22	[0.64–2.35]	0.540	1.35	[0.77–2.38]	0.290
Preoperative FEV1	1.01	[0.99–1.03]	0.240	0.99	[0.97–1.01]	0.320
Preoperative DLCO	1.02	[1.00–1.05]	0.060	0.99	[0.97–1.01]	0.270
cTNM stage	2.46	[0.92–6.55]	0.070	2.25	[0.91–5.53]	0.077
Diabetes	1.77	[0.57–5.51]	0.320	0.61	[0.14–2.60]	0.500
Coronary disease	1.84	[0.41–8.23]	0.420	0.70	[0.09–5.19]	0.720
Thoracotomy	2.77	[1.01–7.59]	0.040	2.62	[1.12–6.14]	0.020
ASA score	1.05	[0.45–2.44]	0.910	1.32	[0.65–2.69]	0.440
Operative time	1.00	[0.99–1.01]	0.990	1.00	[0.99–1.01]	0.990
Number of segments removed	0.57	[0.16–2.01]	0.380	1.25	[0.51–3.06]	0.630
Complex segmentectomy	0.68	[0.22–2.16]	0.510	2.56	[1.10–5.93]	0.020
Run	0.65	[0.23–1.85]	0.420	1.75	[0.76–4.05]	0.190
pTNM stage	1.01	[0.34–2.94]	0.990	3.55	[1.53–8.23]	0.003
Hospital length of stay	0.99	[0.90–1.10]	0.900	1.00	[0.93–1.06]	0.890
SUVmax ≥ 6	0.81	[0.22–2.95]	0.750	2.86	[1.20–6.81]	0.017
Compromise segmentectomy	1.73	[0.58–5.19]	0.320	3.52	[1.45–8.53]	0.005
C/T ratio	0.83	[0.31–2.20]	0.700	1.15	[0.50–2.65]	0.740
Maximum NRS for pain	1.07	[0.85–1.34]	0.560	0.93	[0.76–1.14]	0.470

DFS, disease-free survival; OS, overall survival; HR, hazard ratio; 95% CI, 95% confidence interval; C/T ratio, consolidation-to-tumor ratio; NRS, numeric rating scale; Run, uncertain resection.

The results of overall survival and disease-free survival in multivariable analysis are presented in Table 3. Given the limited number of recurrence and death events, these models were interpreted cautiously.

**Table 3:** Multivariable results for disease-free survival and overall survival.

Characteristic	DFS HR	DFS 95% CI	DFS *p*-value	OS HR	OS 95% CI	OS *p*-value
Sex (male)	5.36	[1.07–26.91]	0.042	2.95	[0.83–10.47]	0.094
pTNM stage	0.89	[0.21–3.69]	0.871	1.06	[0.33–3.38]	0.910
Compromise segmentectomy	0.66	[0.10–4.28]	0.668	3.47	[1.16–10.41]	0.026
SUVmax ≥ 6	0.47	[0.10–2.32]	0.356	2.20	[0.86–5.60]	0.098
Thoracotomy	5.40	[1.51–19.24]	0.009	1.70	[0.65–4.46]	0.280
Complex segmentectomy	0.61	[0.14–2.76]	0.524	2.03	[0.78–5.28]	0.150

DFS, disease-free survival; OS, overall survival; HR, hazard ratio; 95% CI, 95% confidence interval.

**Figure 1:**
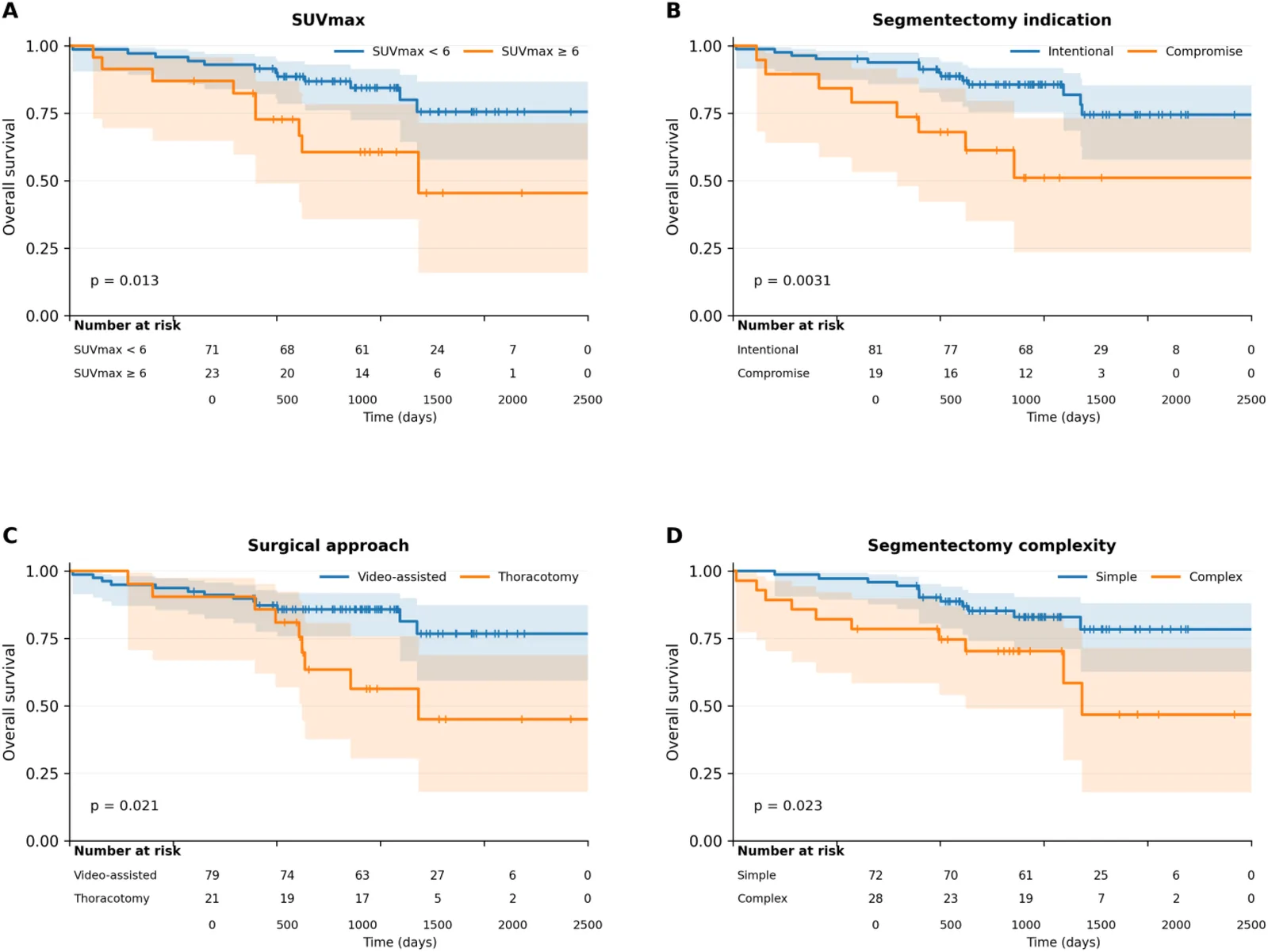
Kaplan—Meier curves for overall survival. **A**: SUVmax < 6 versus SUVmax ≥ 6. **B**: Intentional versus compromise segmentectomy. **C**: Video-assisted approach versus thoracotomy. **D**: Simple versus complex segmentectomy.

**Figure 2:**
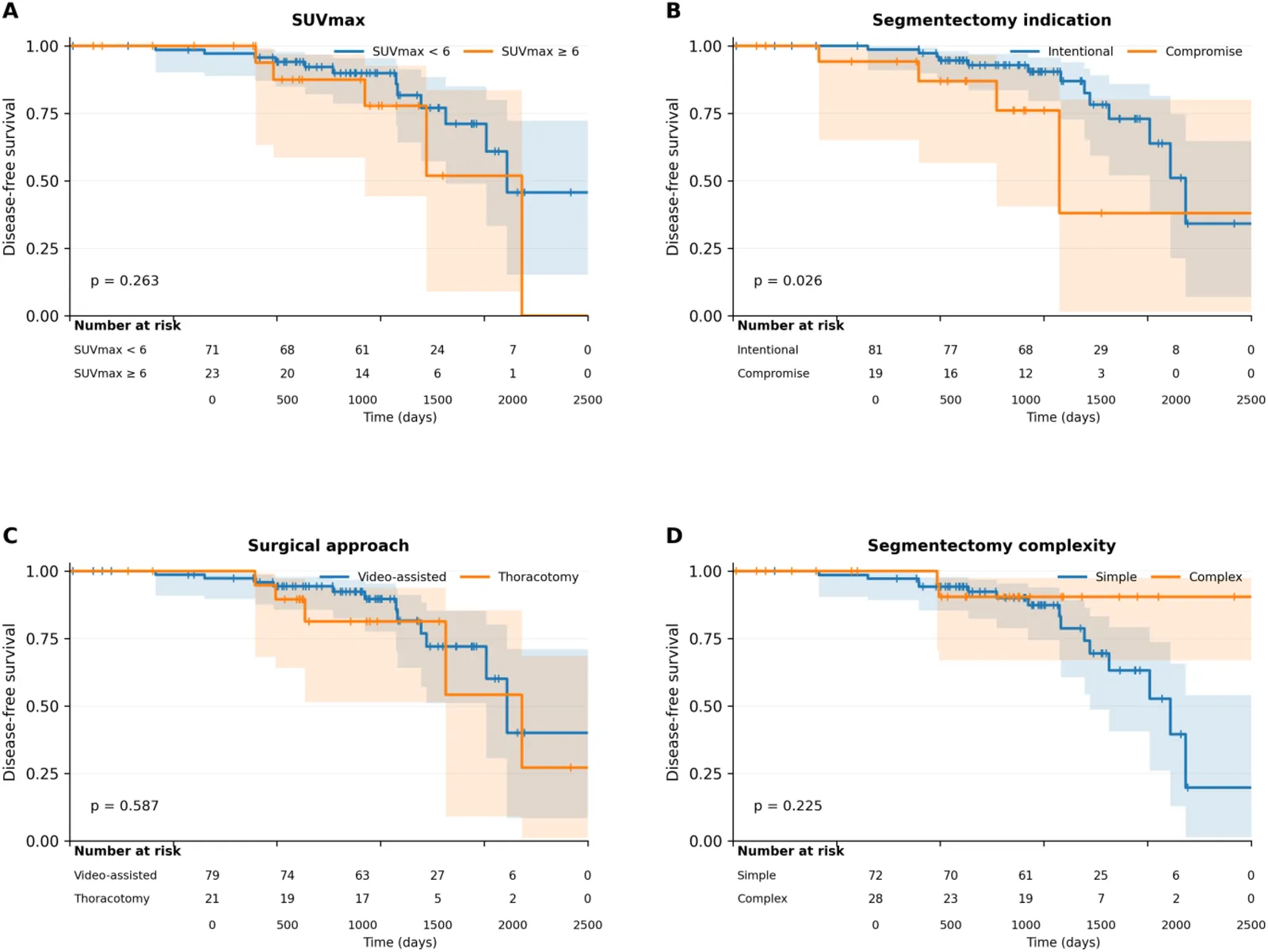
Kaplan—Meier curves for disease-free survival. **A**: SUVmax < 6 versus SUVmax ≥ 6. **B**: Intentional versus compromise segmentectomy. **C**: Video-assisted approach versus thoracotomy. **D**: Simple versus complex segmentectomy.

Disease-free survival and overall survival curves are shown in Figures 1 and 2.

## 4 Discussion

In our study, compromise segmentectomy was associated with worse overall survival in both univariate analysis (HR 3.52; 95% CI 1.45–8.53; *p* = 0.005) and multivariable analysis (HR 3.47; 95% CI 1.16–10.41; *p* = 0.026). In contrast, compromise segmentectomy was not significantly associated with DFS in the univariate Cox model (HR 1.73; 95% CI 0.58–5.19; *p* = 0.320), although a difference was observed on Kaplan–Meier analysis (*p* = 0.026). This discrepancy should be interpreted cautiously and considered exploratory, because the number of events was limited and the confidence intervals were wide. Among the 19 patients who underwent compromise segmentectomy, 8 died, whereas only 4 experienced disease recurrence. These findings suggest that compromise segmentectomy mainly identifies a frail, high-risk patient subgroup with impaired overall survival, rather than demonstrating a clear excess recurrence risk or definite failure of oncologic control. The mean follow-up duration for this subgroup was 43.6 months, comparable to that of the overall cohort, which further supports the possibility of acceptable oncologic disease control in selected patients.

Thoracotomy was also associated with poorer DFS and OS in univariate models (HR 2.77; 95% CI 1.01–7.59; *p* = 0.040 and HR 2.62; 95% CI 1.12–6.14; *p* = 0.020, respectively). In the survival analyses, the comparison was performed between thoracotomy and video-assisted approaches, the latter combining VATS and video-assisted mini-thoracotomy. The poorer outcomes observed after thoracotomy may be explained by both the higher morbidity associated with open surgery and selection of patients with more advanced or technically challenging disease that was not amenable to a video-assisted approach (7).

An SUVmax ≥ 6 was associated with worse OS in univariate analysis (HR 2.86; 95% CI 1.20–6.81; *p* = 0.017), but this association was not statistically significant in the multivariable model (HR 2.20; 95% CI 0.86–5.60; *p* = 0.098). No effect of this variable was observed on DFS (HR 0.81; 95% CI 0.22–2.95; *p* = 0.750). SUVmax is a relative marker of metabolic activity and may reflect either more aggressive tumor biology or reduced baseline metabolism in frail patients. Kocaman et al. (5) demonstrated that high SUVmax was associated with recurrence and mortality and suggested that lobectomy may be superior to segmentectomy in patients operated on for stage I lung cancer. This parameter should probably be considered alongside tumor size when discussing segmentectomy.

We also observed increased mortality in univariate models among patients who underwent complex segmentectomy (HR 2.56; 95% CI 1.10–5.93; *p* = 0.020). This effect, which could partly be explained by reduced resection margins (8), has not been consistently demonstrated in previous series (3, 9). Furthermore, the absence of a significant effect on recurrence and in the multivariable model suggests that the association with mortality may again reflect selection of more fragile patients rather than a direct oncologic effect of resection complexity.

In our study, the consolidation-to-tumor ratio did not impact OS or DFS, although it has previously been described as a prognostic factor of malignancy and aggressiveness (4, 10). Other recurrence risk factors described in the literature but not explored in our study include resection margins smaller than tumor size (11), visceral pleural invasion, and spread through air spaces (STAS) (12), the impact of which remains debated in the setting of segmentectomy.

The central finding of this study is the impaired overall prognosis observed in patients undergoing compromise segmentectomy. Segmentectomy is now recognized as a safe procedure (13, 14) and is increasingly integrated into clinical practice (15), but its indications remain limited. In an increasingly elderly and comorbid population, and in patients with multiple lesions requiring treatment, compromise segmentectomy outside standard indications may represent a pragmatic lung-sparing strategy. Although the functional benefit of segmentectomy over lobectomy may be modest (16), preserving lung parenchyma may be important in patients requiring multisite, sequential, or iterative surgery. To further define the relevance of such compromises, randomized trials or large prospective registries comparing lobectomy, segmentectomy, and possibly stereotactic radiotherapy in polymorbid patients with borderline respiratory function and/or multiple lesions would provide valuable guidance to thoracic oncology teams.

Our study has limitations. First, it was retrospective and single-center, although the longitudinal follow-up allowed excellent data completeness with no patients lost to follow-up. Second, the relatively small number of patients and events (17 recurrences and 22 deaths) limited statistical power and increased the risk of overfitting in multivariable Cox models. Some hazard ratios had wide confidence intervals, and the multivariable results should therefore be interpreted cautiously as exploratory. Third, OS was probably influenced by non-cancer mortality, as only 8 of the 22 deaths were cancer-related. Cancer-specific survival was not analyzed, and detailed causes of non-cancer death were not systematically available. This limitation is particularly relevant when interpreting outcomes in the compromise segmentectomy group, in which baseline frailty and comorbidities likely contributed to mortality. Fourth, although Run status was defined according to the IASLC criteria, the database did not provide a sufficiently reliable itemized breakdown of the reasons for Run classification, such as insufficient lymph node dissection, highest mediastinal node positivity, carcinoma *in situ* at the bronchial margin, or positive pleural cytology. This lack of granularity may explain the absence of a significant association between Run status and outcomes. Nevertheless, to our knowledge, this study reflects real-world practice in a single center within an unselected patient population including compromise segmentectomies.

## 5 Conclusions

This real-world study showed that compromise segmentectomy was associated with impaired overall survival after segmentectomy for NSCLC. This finding appears to identify a frail, high-risk subgroup rather than a clear increase in recurrence or oncologic failure. Thoracotomy, SUVmax ≥ 6, and complex segmentectomy were also associated with mid-term mortality in univariate analyses. Further studies are needed to determine the optimal management strategy for patients who fall at the margins of standard indications for segmentectomy.

## Data Availability

The original contributions presented in this study are included in the article. Further inquiries can be directed to the corresponding author.
